# Exercised-Induced Hypoalgesia following An Elbow Flexion Low-Load Resistance Exercise with Blood Flow Restriction: A Sham-Controlled Randomized Trial in Healthy Adults

**DOI:** 10.3390/healthcare10122557

**Published:** 2022-12-16

**Authors:** Stefanos Karanasios, Alexia Sozeri, George A. Koumantakis, George Gioftsos

**Affiliations:** 1 Laboratory of Advanced Physiotherapy (LAdPhys), Physiotherapy Department, School of Health and Care Sciences, University of West Attica, 12243 Aigaleo, Greece; 2 Physiotherapy Department, School of Health and Care Sciences, University of West Attica, 12243 Aigaleo, Greece

**Keywords:** pressure pain threshold, blood flow restriction, pain sensitivity, resistance exercise

## Abstract

We aimed to evaluate the hypoalgesic effect of an elbow flexion low-load resistance exercise with blood flow restriction (LLRE–BFR) when compared to high-load resistance exercise (HLRE) with sham-BFR in healthy individuals. Forty healthy young adults (17 women), with a mean age ± SD: 26.6 ± 6.8 years, and mean body mass index ± SD: 23.6 ± 2.7 were randomly assigned to either an LLRE–BFR (30% 1 repetition maximum, RM) or an HLRE with sham-BFR group (70% of 1 RM). Blood pressure and pressure pain thresholds (PPTs) were measured pre- and post-exercise intervention. The rating of perceived exertion (RPE) was recorded after each set. There were non-significant between-group changes in PPT at the dominant biceps (−0.61, 95%CI: −1.92 to 0.68) with statistically significant reductions between pre- and post-exercise in LLRE–BFR (effect size, d = 0.88) and HLRE-BFR (effect size, d = 0.52). No within- or between-group differences were recorded in PPT at non-exercising sites of measurement. No mediating effects of changes in blood pressure or RPE on the changes in pressure pain threshold were observed. LLRE–BFR produced a similar hypoalgesic effect locally compared to HLRE and can be used as an alternative intervention to decrease pain sensitivity when HLRE is contraindicated or should be avoided.

## 1. Introduction

Exercise-induced hypoalgesia (EIH) is a widely studied phenomenon that describes an acute reduction in pain perception during or after exercise [1,2]. Evidence suggests that clinically important reductions in pain perception can be observed after a single bout of exercise (ranging from 1 session to 12 weeks) in individuals with and without pain [3,4]. The increases in pain thresholds have been recorded both at local and remote sites lasting from 5′ to 1 h depending on the type of exercise and the modality of the pain test stimulus [4,5,6]. Changes in pain sensitivity are usually measured with quantitative sensory testing including various noxious stimulus (i.e., pressure, thermal and electrical) [4].

Several mechanisms have been suggested to cause EIH including central descending opioid and cannabinoid inhibitory pain pathways [7,8], conditioned pain modulation [9,10,11], the recruitment of high-threshold motor units [5,12], baroreceptor-related mechanisms [13], ischemic and stress-induced hypoalgesic mechanisms [12,14] and psychological contributing factors [15]. Although the exact role and magnitude of each underlying mechanism remains unclear, the analgesic effect of exercise is considered a key clinical tool for the management of different pain conditions [4]. Some critical factors found to influence the magnitude of EIH include the exercise parameters (i.e., type, dose, and intensity) [16,17] and the individual’s characteristics (i.e., age, sex, chronicity and severity) [10,17,18]. It is also well documented that higher-intensity and prolonged exercise can cause greater EIH with higher changes observed in the exercising limb compared to remote sites of the body [19,20]. Research evidence suggests that using either isometric or concentric–eccentric exercises (>65% one repetition maximum-1 RM) can significantly reduce pain sensitivity in individuals with chronic pain presenting a moderate to large hypoalgesic effect depending on the patient population [17]. However, using high-load training in the clinical setting is sometimes contraindicated (post-operated patients, fractures, etc.) or should be avoided. Moreover, some individuals with chronic painful conditions and high severity may present a hyperalgesic affect after training in the exercising limb rather than a decreased pain sensitivity [17,21].

Recently, a novel training method using low-load resistance exercises with blood flow restriction (LLRE–BFR) has been proposed to cause a pain-modulation effect in individuals with or without pain [20,22]. LLRE–BFR involves loads between 20% and 40% of 1 RM with partial restriction of the arterial blood flow by placing inflatable air cuffs at the most proximal part of the exercising limb [23]. BFR training has shown significant improvements in muscle strength [24,25,26,27] and muscle growth compared to conventional training [28,29,30], suggesting that localized hypoxia may provide a significant metabolic stimulus with increased cellular swelling and type II muscle fiber recruitment during exercise [31]. Interestingly, similar physiological responses and benefits have been reported with systemic hypoxia training, which includes multi-joint exercises using hypoxic chambers. [31]. LLRE–BFR has also been shown to produce an acute EIH effect in healthy individuals [19] or significant pain reductions in different pathological populations [32,33,34,35]. Based on a cross-over trial, LLRE–BFR produced a larger hypoalgesic effect compared with the same exercise without BFR and a similar effect compared with a high-load exercise (70% 1 RM) [19]. In the same vein, patients with anterior knee pain reported better outcomes in pain intensity using LLRE–BFR compared with exercise alone, lasting for at least 45′ post-intervention [34]. Recently, another study reported similar EIH following LLRE until failure with or without BFR in healthy individuals, suggesting that BFR can help to achieve the same magnitude of EIH with fewer repetitions [35]. 

Despite the multiple research reports on the effect of LLRE–BFR on EIH, further examination of the optimal exercise parameters is required in order to conclude if it can serve as a pain management intervention. For example, previous reports have only evaluated the effects of lower-limb exercises with BFR (i.e., leg press and knee extension exercises) [19,20,34,36]. Additionally, using higher BFR pressures (80% of limb occlusive pressure, LOP) resulted in a greater increase in the pressure pain thresholds at the exercising limb. [19] However, higher occlusion rates in the upper limb are usually intolerable and may increase the risk of adverse effects [37,38]; therefore, lower pressures (<50% LOP) are recommended [22,38]. 

Based on our current knowledge, studies investigating the effects of dynamic upper-limb low-load exercises with BFR on pain sensitivity are not available. Hence, the primary aim of this randomized sham-controlled trial was to compare the effect of elbow flexion LLRE–BFR to elbow flexion HLRE with sham-BFR on pressure pain thresholds at local and remote sites in healthy individuals. We also sought to investigate if changes in blood pressure and rating of perceived exertion (RPE) mediate the EIH (within-participant mediation analyses) when using LLRE–BFR or HLRE with sham-BFR interventions. 

## 2. Materials and Methods

### Study Design

**Figure 1:**
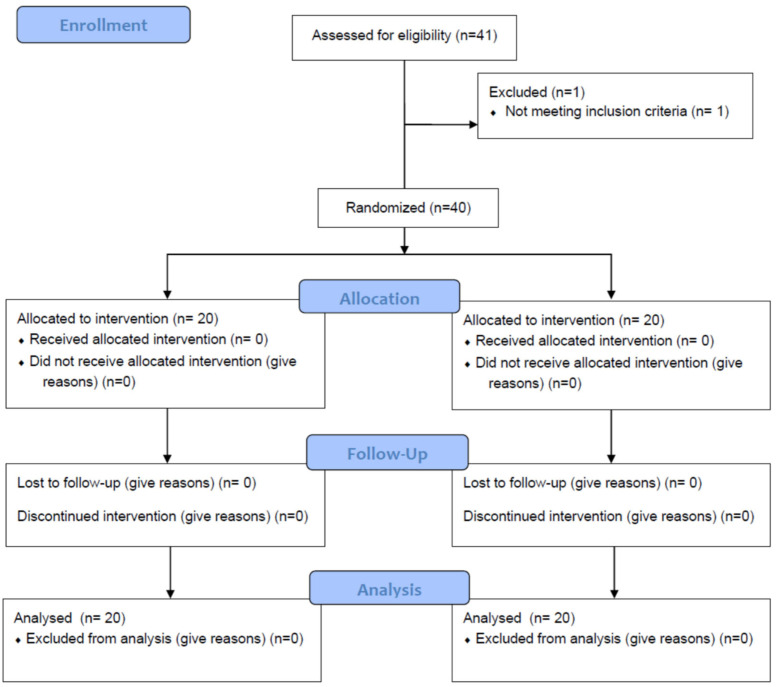
CONSORT statement flow diagram. Abbreviations: BMI, body mass index.

The present study used a prospective randomized sham-controlled design and was conducted in a community setting (Attica, Greece). Participants were recruited from July 2022 to August 2022 via electronic invitations at the University of West Attica. One physiotherapist with 10 years of experience delivered both interventions in a private physiotherapy clinic. The trial was prospectively registered on clinicaltrials.gov (NCT05446103). We adhered to the Consolidated Standards of Reporting Trials (CONSORT) recommendations for designing and reporting the present study (Figure 1).

## 3. Participants

We enrolled 40 healthy young adults between 18 and 40 years old. Other inclusion criteria were a body mass index (BMI) ≤ 30, able to perform full elbow flexion/extension and without neurological, musculoskeletal, or cardiovascular problems. Exclusion criteria were shoulder tendinopathy, cervical radiculopathy, rheumatoid arthritis, neurological deficit, serious cardiovascular diseases, venous deficiency, lymphoedema, history of heart surgery, cancer history, previous breast surgery, orthopedic surgeries < 6 months, thrombosis, diabetes, BMI > 30, Crohn syndrome, cognitive or psychiatric disorders, and family or personal history of pulmonary embolism. Participants were asked to avoid high-intensity activity, caffeine, or alcohol for at least 24 h before measurements. Prior to entering the study, all subjects signed an informed consent form in compliance with the standards of the Declaration of Helsinki [39]. Ethical approval was granted from the University of West Attica Ethics Committee (54099/09-06-2022). 

### 3.1. Randomization and Blinding

A computer-generated randomization sequence at a 1:1 ratio was performed by a researcher who was not involved in data collection. Allocations were sealed in opaque, consecutively numbered envelopes. An independent member of the research team asked participants to pick from the envelopes without looking. All participants and study personnel (assessor and data analyst) were blinded to the group allocation throughout the study. The physiotherapist (AS) who performed the intervention was not involved in the inclusion process or the evaluation of the patient. However, due to the nature of the intervention, the physiotherapist delivering the exercise was not blinded to the group allocation. Initial assessment for eligibility and outcome assessment were conducted by a physiotherapist with 17 years of experience (SK).

### 3.2. Experimental Design

Before randomization, the blinded assessor (SK) recorded demographic characteristics (age, BMI and dominant side), blood pressure and pressure pain thresholds. Participants were asked to rest in a sitting position for 10 min and the non-dominant arm was used for the blood pressure measurement [40]. Then, participants were familiarized with procedures to be used for testing pressure pain threshold at local and remote sites. The measurements of pressure pain threshold were taken three times for each point 5 min prior to exercise and the average value was recorded. Next, participants visited another room and were familiarized with the elbow extension exercise; subsequently, the elbow flexion 1 RM was measured. 

Prior to commencing each session, LOP was determined in the position used for the exercise intervention (standard anatomical position) [40]. Participants were randomized to either an LLRE–BFR group using 40–50% of LOP or an HLRE with sham-BFR group using <20% of LOP (the cuff deflated to the point that was comfortably positioned on the proximal site of the upper limb) [32]. The automatic personalized tourniquet system Mad-Up Pro (France) was used for all BFR applications. Immediately after completing the exercise intervention, participants again visited the assessor’s room where blood pressure and pressure pain thresholds were measured (5 min post intervention) [19,20,36].

## 4. Interventions

Participants in both groups performed an open kinetic chain elbow flexion exercise in the standard anatomical position with equal time under load (1 s concentric, 1 s eccentric phase, paced by a metronome). The exercise included 4 sets of 75 repetitions (30-15-15-15) at 30% 1 RM in the LLRE–BFR group and 4 sets of 10 repetitions at 70% 1 RM in the HLRE with sham-BFR group. A 30 s break was used between sets. The cuff was deflated between sets in the LLRE–BFR group. The physiotherapist motivated all participants to successfully complete all repetitions at each set. If a participant was incapable of finishing two consecutive repetitions or failed to maintain the pacing, then the load was accordingly reduced. Exercise volume (kg) was calculated as: repetitions × exercise load (kg).

### Elbow Flexion One-Repetition Maximum 

To determine 1 RM, participants were asked to warm up by performing 5–10 repetitions of the elbow flexion exercise using 15–20% of the predicted 1 RM [19]. Then, the load was set at 80% of the predicted 1 RM. Following each successful repetition, the load was increased by 0.5 to 1 kg until subjects failed to execute the exercise through the entire range of motion, used improper form to complete the repetition, needed assistance or reported pain [41,42]. Subsequently, the load was decreased, and 1 RM was determined when a participant was able to successfully lift the greatest load one time. Participants rested for 1–2 min between each attempt to ensure recovery [41]. Four to eight attempts were needed to obtain 1 RM for all participants.

## 5. Blood Pressure

Before intervention, participants rested for 10 min in a seated position and blood pressure was measured at the brachial artery of the non-dominant side using an electronic device (Omron M6 Comfort HEM-7321). Systolic and diastolic blood pressures were recorded using the average of two measurements with a 30 s rest between them [40,43]. Blood pressure was also recorded after completing the fourth set of the exercise intervention.

## 6. Pressure Pain Threshold

To measure pressure pain threshold, participants were seated with both arms resting on the thighs and the hips flexed at 90°. Pressure pain thresholds were measured using a 1 cm diameter hand-held digital algometer (Baoshishan ZP-1000 N 20/22806, China). The sequence of measurements was the same pre- and post-intervention starting from the dominant to the non-dominant side in the following order: the middle of the biceps brachii muscle (10 cm proximal to the cubital fossa) [19,44], the lateral epicondyle [45], the upper trapezius muscle (10 cm from the acromion in direct line with the neck) [46] and the middle of the quadriceps muscles (20 cm proximal to the base of the patella) [19]. 

The same blinded assessor (SK) conducted all the measurements using a metronome pacing the rate of pressure application. Participants were instructed to give a verbal notification (“now”) when the stimulation became painful. Three assessments were conducted at each site, with 20–30 s rest between administrations, and the mean values were recorded as kg/cm^2^. We used the familiarization of testing the pressure pain thresholds process (2 measurements with 10 min interval between administrations) to calculate intra-rater reliability using intraclass correlation coefficients, standard error of measurement and minimum detectable change. The results are shown in Supplementary Table S1.

## 7. Rating of Perceived Exertion (RPE)

The Borg RPE 6–20 scale was used to measure RPE, which is a simple self-administrative tool for measuring an individual’s effort and exertion during exercise with excellent reliability and validity in different populations and conditions [47,48,49]. Participants were instructed to rate their effort after each set of exercise if 6 meant they felt no exertion, and 20 meant maximal exertion (the hardest they have ever experienced) [47]. 

## 8. Sample Size

Pressure pain threshold was used as the primary outcome measure to calculate the sample size with GPower 3.1 (UCLA Statistics, Statistical Consulting Center, Los Angeles, CA, USA). The effect size was based on previous reports [50,51] using exercise interventions in patients with upper-limb dysfunctions and healthy individuals measuring pressure pain thresholds. A sample size of 17 in each group was estimated to be sufficient to detect an effect size of 1.0 on pressure pain threshold (power 0.80, two-sided significance level 0.05). To allow for a 10% loss to follow-up, the sample size was increased to 20 per group.

## 9. Statistical Analysis

The normality of the data was visually checked with Q-Q plots and statistically tested using the Shapiro–Wilk test. Descriptive statistics were used to summarize the baseline characteristics of the participants and the measurements. Data were analyzed for between-group differences using mixed-effects models with participant-specific random effects over the two measurement time points (pre- and post-intervention). The fixed effects included group, time, and group × time interactions. The parameter estimates were adjusted for covariates such as sex, age, body mass index, blood pressure and dominant side. The choice of the best model for each variable was made on the basis of the Akaike information criterion. Where a significant main effect or interaction was found, we performed post-hoc testing. Adjustments were performed for post-hoc comparisons (Bonferroni). RPE was assessed using repeated-measures ANOVA with condition (LLRE–BFR and HLRE with sham-BFR) and time (set 1–4) as within-subject factors (Bonferroni corrections). Cohen’s d effect sizes were calculated by using the pooled standard deviations (SDs) of the baseline scores, and values of 0.2, 0.5, and 0.80 were considered the thresholds of a small, moderate, and large effect, respectively [52]. All data were analyzed with IBM SPSS (Version 25) (IBM Corp., Armonk, NY, USA) and the level of significance was set at 0.05.

We conducted a within-participant mediation analysis using the SPSS macro “MEMORE” to investigate the effect of the changes in blood pressure or the exercise-induced RPE in the relationship between exercise intervention and changes in pressure pain threshold. The type of intervention (LLRE–BFR and HLRE with sham-BFR) was the independent variable (X), the changes in pressure pain threshold were the dependent variable (Y), the changes in blood pressure were Mediator 1 and the exercise-induced RPE was Mediator 2 [53]. Bootstrapping methods involving randomly resampling the data 5000 times were used to calculate the 95% confidence intervals (95%CIs) of the coefficients for the total, direct and indirect effect (a*b) [53]. All statistical analyses were conducted for each site separately.

## 10. Results

Forty healthy individuals (17 women and 23 men) completed all experimental procedures. The mean age (±SD) of the participants was 26.6 years (±6.8) with a mean BMI (±SD) of 23.6 (±2.7) (Table 1). Summary statistics of baseline-adjusted values of pressure pain thresholds and blood pressure measurements by treatment group are presented in Table 2 and Supplementary Figures S1a–h. 

## 11. Between-Group Differences

There were non-significant differences between the LLRE–BFR and HLRE with sham-BFR groups in pressure pain thresholds at the dominant and non-dominant sites of the biceps (−0.61, 95%CI:−1.92 to 0.68 & 0.02, 95%CI: −1.5 to 1.5, respectively), lateral epicondyle (0.99, 95%CI: −1.1 to 3 &1, 95%CI: −0.9 to 3, respectively), upper trapezius (0.3, 95%CI: −2.1 to 2.8 & 0.19 95%CI: −2.1 to 2.5, respectively) and quadriceps (−0.07 95%CI: −3 to 2.8 & −0.08, 95%CI: −2.4 to 2.6, respectively) post-intervention (Table 2). 

There were non-significant differences in systolic (−7.4, 95%CI: −21.7 to 6.8) and diastolic (−1.57, 95%CI: −8.1 to 4.9) blood pressure between groups as measured immediately after the interventions (Table 2). The RPE increased after each set of exercise for both groups (*p* < 0.05) (Table 3). There was no significant interaction between the time and type of intervention in the RPE (F = 0.727, *p* = 0.538). A statically significant difference in the RPE in favor of HLRE compared to LLRE–BFR was recorded after set 2 while no differences were found between the groups after sets 1, 3 and 4 (Table 3).

Four participants (3 men and 1 woman) from the LLRE–BFR group and three (1 man and 2 women) from the HLRE with sham-BFR group reported increased muscle pain (delayed-onset muscle soreness, DOMS) in the exercising limb 24 h after exercise. 

## 12. Mediation

Mediation analysis resulted in no significant effect of the change in systolic blood pressure (Mediator 1), nor RPE (Mediator 2) on the changes in pressure pain threshold (Y) between the exercise groups (X) at the local or distal sites of measurement (Supplementary Table S2).

## 13. Within-Group Differences 

Statistically significant within-group changes between pre- and post-exercise were found for the LLRE–BFR (−1.61, 95%CI: −3.14 to 0.07; *p* < 0.001) and HLRE with sham-BFR (−0.95, 95%CI: −1.71 to −0.79; *p* = 0.016) groups in pressure pain thresholds at the dominant biceps. There were non-significant within-group changes in the pressure pain thresholds at the rest sites of measurements and the systolic/diastolic blood pressure in both groups between pre- and post-exercise (Supplementary Table S3). 

## 14. Discussion

To our knowledge, this is the first study investigating the effect of an upper-limb exercise using low-load resistance with BFR compared to high-load resistance without BFR in pain sensitivity. The main finding of our study suggests that a single elbow flexion LLRE–BFR produces a similar hypoalgesic effect at a local site as compared with the same HLRE. However, non-significant within- or between-group changes were observed at non-exercising sites of measurement. Additionally, blood pressure changes and the RPE were not found to mediate the relationship between exercise intervention and changes in pressure pain thresholds at the exercising limb. Both exercise groups presented a similar rate of adverse events (DOMS).

### 14.1. Low-Load Resistance Exercise with BFR Can Be An Alternative Intervention to Decrease Pain Sensitivity

Significant changes in pain sensitivity at the exercising limb were observed in both groups. Considering that these changes were not mediated by the RPE or blood pressure changes, the most possible explanations underpinning pain modulation after LLRE–BFR might include the activation of the endogenous opioid system (release of beta-endorphins) or high-threshold motor unit recruitment during exercise [4,7,19,54]. Our findings were in agreement with a previous study using a cross-over design and comparing LLRE–BFR to HLRE following a single leg press exercise in healthy individuals [19]. In contrast, contemporary evidence suggested that exercise-induced hypoalgesia after low-intensity exercise with BFR was not driven by the blood flow restriction component but due to the work volume (exercise to failure) [36,55]. However, these studies did not include a comparison group using high-load exercise interventions, which limits further comparisons to the present results. 

Based on the findings by Hughes et al. [19], LLRE–BFR with low pressure (40% LOP) resulted in similar increases in pressure pain thresholds compared to HLRE. Additionally, LLRE–BFR with high pressure (80%LOP) presented significantly greater ΕΙH compared to the other exercise trials such as LLRE–BFR with low pressure, LLRE and HLRE [19]. Different research reports suggested that BFR exercises with high occlusion pressure (60%−80% LOP) can reduce pain more than HLRE [20,33,34]; however, similar comparisons in the upper limb are considered inappropriate due to the risk of adverse effects when using high BFR pressures [37,38]. 

In our study we adhered to the best BFR practice guidelines that recommend the use of pressures <50% LOP [56,57,58] in the upper limb, resulting in a mean (±SD) tolerable pressure of 41.5% LOP (±2.3). Despite the low occlusion pressure, our findings suggested similar EIH between LLRE–BFR and HLRE with a large within-group effect size (d = 0.88) between pre- and post- intervention. Therefore, the method can be suggested as an alternative useful intervention to decrease pain sensitivity when high-load resistance exercise is contraindicated or should be avoided. 

### 14.2. Elbow Flexion Low-Load Resistance Exercise with BFR Produces Local but Not Remote Hypoalgesia

Based on our results, elbow flexion LLRE–BFR produced only local hypoalgesia with non-significant changes at non-exercising sites between pre- and post-exercise, suggesting that only local segmental mechanisms played a role. Previous reports [19,36] have shown significant increases in pressure pain thresholds after LLRE–BFR at non-exercising sites of the body, indicating that systemic mechanisms are playing a critical role such as CPM due to discomfort and baroreceptor-related mechanisms due to the increase in blood pressure. Opposite to the previous findings, Song et al. (2022) suggested that low-load exercise to failure with or without BFR produces similar hypoalgesic effects at remote sites, which was not mediated by discomfort or changes in blood pressure [36]. Hence, the EIH was mostly explained by the release of beta-endorphins (stimulating group III and IV afferents) and high-threshold motor unit recruitment as well [36,59,60]

A possible explanation for the lack of significant reduction in pain sensitivity at non-exercising sites in our study may be based on the selected exercising limb (upper vs. lower) and the exercise work volume used (failure vs. not to failure). For example, based on previous findings, high- and low-intensity isometric leg exercises presented significantly greater reductions in pain sensitivity at non-exercising sites due to a condition pain modulation mechanism compared to high- and low-intensity isometric arm exercise [61,62]. Most importantly, exercise to failure has been shown to induce a significant reduction in pressure pain sensitivity at local and remote sites irrespective to the use of BFR [36,55]; therefore, the exercise volume used (not to failure) possibly was not enough to reduce pain sensitivity at non-exercising sites for both exercise groups. Nevertheless, further research is required to evaluate if EIH is dependent on the exercising limb (upper or lower), the dose or the work volume (failure or not) following resistance exercises with or without BFR.

### 14.3. Limitations and Future Research

We acknowledge as a limitation that we used only one measure of EIH (mechanical pressure sensitivity); possibly, by including additional measures such as hot and cold sensitivity, baroreceptor activity, or biomarkers (such as beta-endorphin), we could provide more information about EIH and its underlying mechanisms. Although we intended to investigate the hypoalgesic effect of two specific exercise conditions, including a control group could help to precisely evaluate the true effect of these exercise interventions in pain sensitivity. Additionally, taking into account that we included only short-term measurements (5′ post-exercise), it is not clear if EIH after elbow flexion LLRE–BFR or HLRE remains for longer periods of time.

Our sample consisted of young healthy individuals; therefore, our results cannot be generalized in chronic pain populations. Further research to examine the hypoalgesic effects of LLRE–BFR in patients with chronic pain is necessary.

## 15. Conclusions

The present study demonstrated that an elbow flexion LLRE–BFR produces a similar short-term reduction in pain sensitivity at local sites compared to an elbow flexion HLRE with sham-BFR in healthy individuals. Considering that non-significant changes in pain sensitivity were observed at non-exercising sites and the results of our mediation analysis, this hypoalgesic effect was likely driven by local (segmental) and not systemic (central) mechanisms. Further research is necessary to investigate the effects of upper-limb LLRE–BFR using different doses in healthy individuals and chronic pain populations.

## Figures and Tables

**Table 1:** Participants’ baseline characteristics and exercise parameters in the LLRE–BFR and the HLRE with sham-BFR group.

Characteristics	LLRE–BFR Group (*n* = 20)	LLRE with Sham-BFR Group (*n* = 20)
Women	8 (40%)	9 (45%)
Men	12(60%)	11 (55%)
Age, mean ± SD, years	27.8 ± 6.8	25.4 ± 6.7
BMI, mean ± SD	23.2 ± 1.7	24 ± 3.4
1 RM	15.5 ± 6.2	14.36 ± 6
Work volume	348.37 ± 116.6	367.26 ± 139.2
Exercising Pressure	41.5% ± 2.3	-

Notes: Values are presented as number (percentage), unless otherwise indicated. No significant differences were found between the groups at baseline (all *p* > 0.05). Abbreviations: LLRE–BFR; low-load resistance exercise with blood flow restriction; SD, standard deviation.

**Table 2:** Between-group differences of baseline-adjusted values of pressure pain thresholds and blood pressure.

	LLRE–BFR Group *	HLRE with Sham-BFR Group *	Between-Group Differences ^†^	*p*-Value
**Dominant Biceps**				0.52 ^₸^
Pre-	4.83 ± 1.9 (3.95 to 5.81)	4.87 ± 2.07 (3.95 to 5.89)	-	-
Post-	6.44 ± 1.9 (5.53 to 7.36)	5.83 ± 1.9 (4.91 to 6.74)	−0.61 (−1.92 to 0.68), d = 0.33	0.34 ^¶^
**Non-dominant Biceps**				0.63 ^₸^
Pre-	4.74 ± 1.9 (3.8 to 5.6)	5.15 ± 1.9 (4.2 to 6)	-	-
Post-	5.79 ± 1.8 (4.9 to 6.6)	5.8 ± 1.9 (4.9 to 6.7)	0.02 (−1.5 to 1.5)	1 ^¶^
**Dominant LE**				0.6 ^₸^
Pre-	5.26 ± 2.6 (4 to 6.5)	5.68 ± 1.9 (4.4 to 6.8)	-	-
Post-	5.9 ± 2.6 (4.6 to 7.1)	6.9 ± 2.6 (5.6 to 8.1)	0.99 (−1.1 to 3)	0.6 ^¶^
**Non-dominant LE**				0.61 ^₸^
Pre-	5.35 ± 2.5 (4.1 to 6.5)	5.8 ±2.3 (4.7 to 6.9)	-	-
Post-	5.5 ± 2.4 (4.3 to 6.6)	6.5 ± 2.4 (5.3 to 7.6)	1 (−0.9 to 3)	0.53 ^¶^
**Dominant UT**				0.54 ^₸^
Pre-	6.9 ± 3.2 (5.4 to 8.4)	8.1 ± 3 (6.6 to 9.5)	-	-
Post-	8 ± 3(6.5 to 9.4)	8.3 ± 2.6 (6.8 to 9.8)	0.3 (−2.1 to 2.8)	0.98 ^¶^
**Non-dominant UT**				0.75 ^₸^
Pre-	6.8 ± 2.9 (5.4 to 8.2)	7.41 ± 2.2 (6 to 8.7)	-	-
Post-	8.17 ± 2.9 (6.7 to 9.5)	8.36 ± 2.8 (6.9 to 9.7)	0.19 (−2.1 to 2.5)	0.99 ^¶^
**Dominant Quadriceps**				0.83 ^₸^
Pre-	11.1 ± 3.7 (9.4 to 12.9)	10.77 ± 3.7 (9 to 12.5)	-	-
Post-	10.7 ± 3.7 (9 to 12.5)	10.77 ± 3.6 (8.9 to 12.4)	−0.07 (−3 to 2.8)	0.99 ^¶^
**Non-dominant Quadriceps**				0.8 ^₸^
Pre-	10.5 ± 3.2 (9 to 12)	10.9 ± 3.3 (9.4 to 12.5)	-	-
Post-	11.19 ± 3 (9.7 to 12.6)	11.2 ± 3.3 (9.7 to 12.8)	−0.08 (−2.4 to 2.6)	0.99 ^¶^
**Systolic Blood Pressure**				0.2 ^₸^
Pre-	119.4 ± 9.7 (110.9 to 120)	121.7 ± 17.6 (113.4 to 129.9)	-	-
Post-	124.3 ± 17.8 (116 to 132.7)	116.9 ± 18.4 (108.6 to 125.9)	−7.4 (−21.7 to 6.8)	0.5 ^¶^
**Diastolic Blood Pressure**				0.88 ^₸^
Pre-	75.9 ± 8.3 (72 to 79.8)	74.8 ± 8.1 (71 to 78.6)	-	-
Post-	76.3 ± 8.1 (72.5 to 80.1)	74.76 ± 8.1 (70.9 to 78.5)	−1.57 (−8.1 to 4.9)	0.92 ^¶^

* Values are means ± SD and 95% confidence intervals; ^†^ Values in parentheses are 95% confidence intervals; ^₸^ intervention; ^¶^ Adjustments were performed for post-hoc multiple comparisons (Bonferroni); Abbreviations: BFR, blood flow restriction; LLRΕ–BFR; low-load resistance exercise with blood flow restriction; LE, lateral epicondyle; UT, upper trapezius.

**Table 3:** Outcomes and between-group differences in rating of perceived exertion after each set of exercise.

	Group	Mean ± SD	HLRE with Sham-BFR—LLRE–BFR *	*p* Value
Set 1	HLRE with sham-BFR	13.4 ± 2.7	0.7 (−0.97 to 2.37)	0.404
	LLRE–BFR	12.7 ± 2.4		
Set 2	HLRE with sham-BFR	15.6 ± 2.4	1.9 (0.3 to 3.4)	0.02
	LLRE–BFR	13.7 ± 2.5		
Set 3	HLRE with sham-BFR	16.1 ± 2	1.4 (−0.11 to 2.9)	0.07
	LLRE–BFR	14.7 ± 2.6		
Set 4	HLRE with sham-BFR	16.6 ± 1.5	1.35 (−0.1 to 2.8)	0.075
	LLRE–BFR	15.25 ± 2.9		

* Values in parentheses are 95% confidence intervals; Abbreviations: BFR, blood flow restriction; LLRΕ–BFR; low-load resistance exercise with blood flow restriction.

## Data Availability

The data are available upon request from the corresponding author.

## Supplementary Materials

The following supporting information can be downloaded at: https://www.mdpi.com/article/10.3390/healthcare10122557/s1, Figure S1: Graphical representation of mean pressure pain thresholds (kg/cm^2^) at local and remote sites of measurements (pre- and post-intervention); Table S1: Baseline pressure pain thresholds values with intra-rater reliability using intraclass correlation coefficients, standard error of measurement and minimum detectable change at each site; Table S2: Coefficients, confidence intervals, and significance statistics for within-participant mediation analyses between exercise intervention (“X”), the change in systolic blood pressure (Mediator 1) and RPE (Mediator 2). The outcome (Y) is the change in pressure pain threshold at local and distal sites of measurement. Table S3: Within-group differences (pre-post) of baseline-adjusted values of pressure pain thresholds and blood pressure.

## References

[1] Rice D., Nijs J., Kosek E., Wideman T., Hasenbring M.I., Koltyn K., Graven-Nielsen T., Polli A. (2019). Exercise-Induced Hypoalgesia in Pain-Free and Chronic Pain Populations: State of the Art and Future Directions. J. Pain.

[2] Sluka K.A., Frey-Law L., Hoeger Bement M. (2018). Exercise-induced pain and analgesia? Underlying mechanisms and clinical translation. Pain.

[3] Sandal L.F., Roos E.M., Bøgesvang S.J., Thorlund J.B. (2016). Pain trajectory and exercise-induced pain flares during 8 weeks of neuromuscular exercise in individuals with knee and hip pain. Osteoarthr. Cartil..

[4] Vaegter H.B., Jones M.D. (2020). Exercise-induced hypoalgesia after acute and regular exercise: Experimental and clinical manifestations and possible mechanisms in individuals with and without pain. Pain Rep..

[5] Hoffman M.D., Shepanski M.A., Ruble S.B., Valic Z., Buckwalter J.B., Clifford P.S. (2004). Intensity and duration threshold for aerobic exercise-induced analgesia to pressure pain. Arch. Phys. Med. Rehabil..

[6] Wewege M.A., Jones M.D. (2021). Exercise-Induced Hypoalgesia in Healthy Individuals and People With Chronic Musculoskeletal Pain: A Systematic Review and Meta-Analysis. J. Pain.

[7] Koltyn K.F., Brellenthin A.G., Cook D.B., Sehgal N., Hillard C. (2014). Mechanisms of exercise-induced hypoalgesia. J. Pain.

[8] Starowicz K., Malek N., Przewlocka B. (2013). Cannabinoid receptors and pain. Wiley Interdiscip. Rev..

[9] Koltyn K.F., Knauf M.T., Brellenthin A.G. (2013). Temporal summation of heat pain modulated by isometric exercise. Eur. J. Pain.

[10] Vierck C.J., Staud R., Price D.D., Cannon R.L., Mauderli A.P., Martin A.D. (2001). The effect of maximal exercise on temporal summation of second pain (windup) in patients with fibromyalgia syndrome. J. Pain.

[11] Lemley K.J., Hunter S.K., Bement M.K. (2015). Conditioned pain modulation predicts exercise-induced hypoalgesia in healthy adults. Med. Sci. Sport. Exerc..

[12] Koltyn K.F., Arbogast R.W. (1998). Perception of pain after resistance exercise. Br. J. Sport. Med..

[13] Koltyn K.F., Umeda M. (2006). Exercise, Hypoalgesia and Blood Pressure. Sport. Med..

[14] Pertovaara A., Huopaniemi T., Virtanen A., Johansson G. (1984). The influence of exercise on dental pain thresholds and the release of stress hormones. Physiol. Behav..

[15] Jones M.D., Valenzuela T., Booth J., Taylor J.L., Barry B.K. (2017). Explicit Education About Exercise-Induced Hypoalgesia Influences Pain Responses to Acute Exercise in Healthy Adults: A Randomized Controlled Trial. J. Pain.

[16] Gajsar H., Titze C., Hasenbring M.I., Vaegter H.B. (2017). Isometric Back Exercise Has Different Effect on Pressure Pain Thresholds in Healthy Men and Women. Pain Med..

[17] Naugle K.M., Fillingim R.B., Riley J.L. (2012). A meta-analytic review of the hypoalgesic effects of exercise. J. Pain.

[18] Vaegter H.B., Handberg G., Graven-Nielsen T. (2016). Hypoalgesia After Exercise and the Cold Pressor Test is Reduced in Chronic Musculoskeletal Pain Patients With High Pain Sensitivity. Clin. J. Pain.

[19] Hughes L., Patterson S.D. (2020). The effect of blood flow restriction exercise on exercise-induced hypoalgesia and endogenous opioid and endocannabinoid mechanisms of pain modulation. J. Appl. Physiol..

[20] Song J.S., Spitz R.W., Yamada Y., Bell Z.W., Wong V., Abe T., Loenneke J.P. (2021). Exercise-induced hypoalgesia and pain reduction following blood flow restriction: A brief review. Phys. Ther. Sport.

[21] Chang W.-J., Buscemi V., Liston M.B., McAuley J.H., Hodges P.W., Schabrun S.M. (2019). Sensorimotor cortical activity in acute low back pain: A cross-sectional study. J. Pain.

[22] Patterson S.D., Hughes L., Warmington S., Burr J., Scott B.R., Owens J., Abe T., Nielsen J.L., Libardi C.A., Laurentino G. (2019). Blood Flow Restriction Exercise: Considerations of Methodology, Application, and Safety. Front. Physiol..

[23] Loenneke J.P., Abe T., Wilson J.M., Ugrinowitsch C., Bemben M.G. (2012). Blood flow restriction: How does it work?. Front. Physiol..

[24] Early K.S., Rockhill M., Bryan A., Tyo B., Buuck D., McGinty J. (2020). Effect of Blood Flow Restriction Training on Muscular Performance, Pain and Vascular Function. Int. J. Sport. Phys. Ther..

[25] Takarada Y., Takazawa H., Sato Y., Takebayashi S., Tanaka Y., Ishii N. (2000). Effects of resistance exercise combined with moderate vascular occlusion on muscular function in humans. J. Appl. Physiol..

[26] Cook S.B., Clark B.C., Ploutz-Snyder L.L. (2007). Effects of exercise load and blood-flow restriction on skeletal muscle function. Med. Sci. Sport. Exerc..

[27] Cook C.J., Kilduff L.P., Beaven C.M. (2014). Improving strength and power in trained athletes with 3 weeks of occlusion training. Int J Sport. Physiol Perform.

[28] Farup J., de Paoli F., Bjerg K., Riis S., Ringgard S., Vissing K. (2015). Blood flow restricted and traditional resistance training performed to fatigue produce equal muscle hypertrophy. Scand. J. Med. Sci. Sport..

[29] Yasuda T., Ogasawara R., Sakamaki M., Ozaki H., Sato Y., Abe T. (2011). Combined effects of low-intensity blood flow restriction training and high-intensity resistance training on muscle strength and size. Eur. J. Appl. Physiol..

[30] Yasuda T., Fukumura K., Iida H., Nakajima T. (2015). Effects of detraining after blood flow-restricted low-load elastic band training on muscle size and arterial stiffness in older women. SpringerPlus.

[31] Scott B.R., Slattery K.M., Sculley D.V., Dascombe B.J. (2014). Hypoxia and resistance exercise: A comparison of localized and systemic methods. Sport. Med..

[32] Giles L., Webster K.E., McClelland J., Cook J.L. (2017). Quadriceps strengthening with and without blood flow restriction in the treatment of patellofemoral pain: A double-blind randomised trial. Br. J. Sport. Med..

[33] Hughes L., Rosenblatt B., Haddad F., Gissane C., McCarthy D., Clarke T., Ferris G., Dawes J., Paton B., Patterson S.D. (2019). Comparing the Effectiveness of Blood Flow Restriction and Traditional Heavy Load Resistance Training in the Post-Surgery Rehabilitation of Anterior Cruciate Ligament Reconstruction Patients: A UK National Health Service Randomised Controlled Trial. Sport. Med..

[34] Korakakis V., Whiteley R., Giakas G. (2018). Low load resistance training with blood flow restriction decreases anterior knee pain more than resistance training alone. A pilot randomised controlled trial. Phys. Ther. Sport.

[35] Ferraz R.B., Gualano B., Rodrigues R., Kurimori C.O., Fuller R., Lima F.R., De SÁ-Pinto A.N.A.L., Roschel H. (2018). Benefits of Resistance Training with Blood Flow Restriction in Knee Osteoarthritis. Med. Sci. Sport. Exerc..

[36] Song J.S., Kataoka R., Yamada Y., Wong V., Spitz R.W., Bell Z.W., Loenneke J.P. (2022). The Hypoalgesic Effect of Low-Load Exercise to Failure Is Not Augmented by Blood Flow Restriction. Res. Q. Exerc. Sport.

[37] Counts B.R., Dankel S.J., Barnett B.E., Kim D., Mouser J.G., Allen K.M., Thiebaud R.S., Abe T., Bemben M.G., Loenneke J.P. (2016). Influence of relative blood flow restriction pressure on muscle activation and muscle adaptation. Muscle Nerve.

[38] Kim D., Loenneke J.P., Ye X., Bemben D.A., Beck T.W., Larson R.D., Bemben M.G. (2017). Low-load resistance training with low relative pressure produces muscular changes similar to high-load resistance training. Muscle Nerve.

[39] Association W.M. (2013). World Medical Association Declaration of Helsinki: Ethical Principles for Medical Research Involving Human Subjects. JAMA.

[40] Karanasios S., Koutri C., Moutzouri M., Xergia S.A., Sakellari V., Gioftsos G. (2021). The Effect of Body Position and the Reliability of Upper Limb Arterial Occlusion Pressure Using a Handheld Doppler Ultrasound for Blood Flow Restriction Training. Sport. Health.

[41] Abe T., Kearns C.F., Sato Y. (2006). Muscle size and strength are increased following walk training with restricted venous blood flow from the leg muscle, Kaatsu-walk training. J. Appl. Physiol..

[42] Karanasios S., Korakakis V., Moutzouri M., Xergia S.A., Tsepis Ε., Gioftsos G. (2022). Low-load resistance training with blood flow restriction is effective for managing lateral elbow tendinopathy: A randomized, sham-controlled trial. J. Orthop. Sport. Phys. Ther..

[43] Lacruz M.E., Kluttig A., Kuss O., Tiller D., Medenwald D., Nuding S., Greiser K.H., Frantz S., Haerting J. (2017). Short-term blood pressure variability—Variation between arm side, body position and successive measurements: A population-based cohort study. BMC Cardiovasc. Disord..

[44] Hidalgo-Lozano A., Fernández-de-las-Peñas C., Alonso-Blanco C., Ge H.Y., Arendt-Nielsen L., Arroyo-Morales M. (2010). Muscle trigger points and pressure pain hyperalgesia in the shoulder muscles in patients with unilateral shoulder impingement: A blinded, controlled study. Exp. Brain Res..

[45] Fernández-Carnero J., Binderup A.T., Ge H.Y., Fernández-de-las-Peñas C., Arendt-Nielsen L., Madeleine P. (2010). Pressure pain sensitivity mapping in experimentally induced lateral epicondylalgia. Med. Sci. Sport. Exerc..

[46] Nascimento J.D.S.d., Alburquerque-Sendín F., Vigolvino L.P., Oliveira W.F.d., Sousa C.d.O. (2020). Absolute and Relative Reliability of Pressure Pain Threshold Assessments in the Shoulder Muscles of Participants With and Without Unilateral Subacromial Impingement Syndrome. J. Manip. Physiol. Ther..

[47] Williams N. (2017). The Borg rating of perceived exertion (RPE) scale. Occup. Med..

[48] Lamb K.L., Eston R.G., Corns D. (1999). Reliability of ratings of perceived exertion during progressive treadmill exercise. Br. J. Sport. Med..

[49] Skinner J.S., Hutsler R., Bergsteinová V., Buskirk E.R. (1973). The validity and reliability of a rating scale of perceived exertion. Med. Sci. Sport..

[50] Kuppens K., Struyf F., Nijs J., Cras P., Fransen E., Hermans L., Meeus M., Roussel N. (2016). Exercise- and Stress-Induced Hypoalgesia in Musicians with and without Shoulder Pain: A Randomized Controlled Crossover Study. Pain Physician.

[51] Lannersten L., Kosek E. (2010). Dysfunction of endogenous pain inhibition during exercise with painful muscles in patients with shoulder myalgia and fibromyalgia. Pain.

[52] Cohen J. (1988). Statistical Power Analysis for the Behavioral Sciences.

[53] Montoya A.K., Hayes A.F. (2017). Two-condition within-participant statistical mediation analysis: A path-analytic framework. Psychol. Methods.

[54] Fatela P., Mendonca G.V., Veloso A.P., Avela J., Mil-Homens P. (2019). Blood Flow Restriction Alters Motor Unit Behavior During Resistance Exercise. Int. J. Sport. Med..

[55] Song J.S., Yamada Y., Wong V., Bell Z.W., Spitz R.W., Abe T., Loenneke J.P. (2022). Hypoalgesia following isometric handgrip exercise with and without blood flow restriction is not mediated by discomfort nor changes in systolic blood pressure. J. Sport. Sci..

[56] Centner C., Wiegel P., Gollhofer A., König D. (2019). Effects of blood flow restriction training on muscular strength and hypertrophy in older individuals: A systematic review and meta-analysis. Sport. Med..

[57] Lauber B., König D., Gollhofer A., Centner C. (2021). Isometric blood flow restriction exercise: Acute physiological and neuromuscular responses. BMC Sport. Sci. Med. Rehabil..

[58] Loenneke J.P., Kim D., Fahs C.A., Thiebaud R.S., Abe T., Larson R.D., Bemben D.A., Bemben M.G. (2015). Effects of exercise with and without different degrees of blood flow restriction on torque and muscle activation. Muscle Nerve.

[59] Hirayama A., Saitoh Y., Kishima H., Shimokawa T., Oshino S., Hirata M., Kato A., Yoshimine T. (2006). Reduction of intractable deafferentation pain by navigation-guided repetitive transcranial magnetic stimulation of the primary motor cortex. Pain.

[60] Misra G., Paris T.A., Archer D.B., Coombes S.A. (2014). Dose-Response Effect of Isometric Force Production on the Perception of Pain. PLoS ONE.

[61] Alsouhibani A., Vaegter H.B., Hoeger Bement M. (2019). Systemic Exercise-Induced Hypoalgesia Following Isometric Exercise Reduces Conditioned Pain Modulation. Pain Med..

[62] Vaegter H.B., Handberg G., Graven-Nielsen T. (2014). Similarities between exercise-induced hypoalgesia and conditioned pain modulation in humans. Pain.

