# Supplementary material for: Exercised-Induced Hypoalgesia following An Elbow Flexion Low-Load Resistance Exercise with Blood Flow Restriction: A Sham-Controlled Randomized Trial in Healthy Adults
Source: Healthcare (Basel). 2022 Dec 16;10(12):2557. doi: 10.3390/healthcare10122557 (PMC9778505; doi:10.3390/healthcare10122557)
Supplement: Supplementary file 1 [file healthcare-10-02557-s001.zip › healthcare-2032975-Supplementary Figure S1.pdf]

Figure S1 (a–h). Graphical representation of mean pressure pain thresholds (kg/cm^2) at local and remote sites of measurements (pre- and post-intervention). Error bar is constructed using ±Std Error.

a)

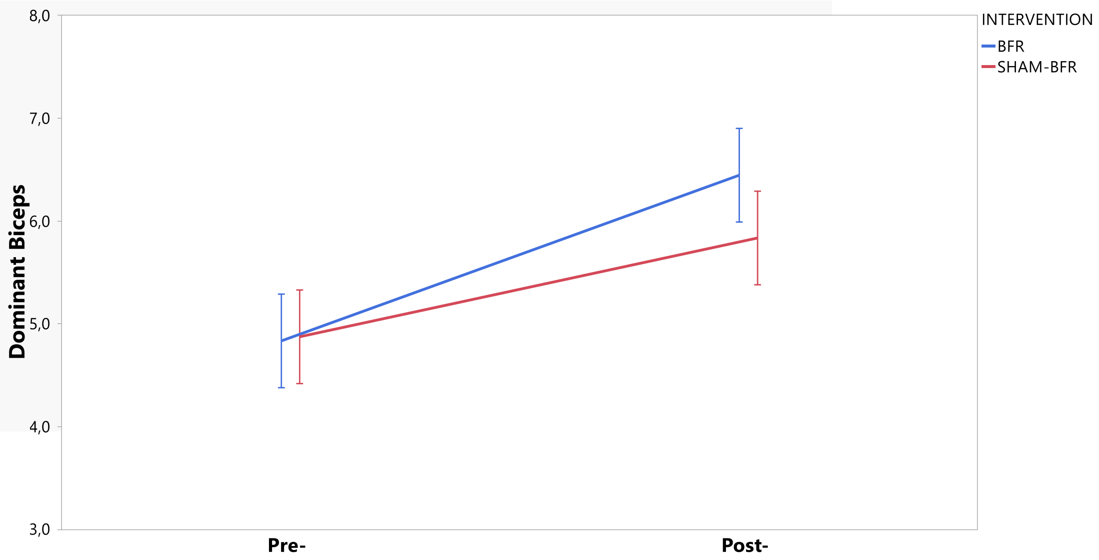

b)

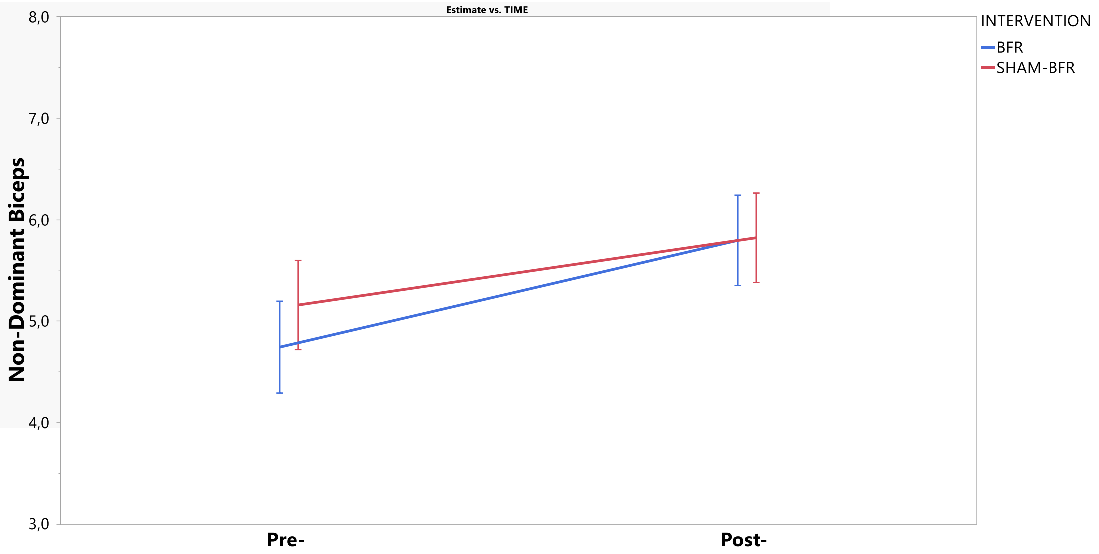

c)

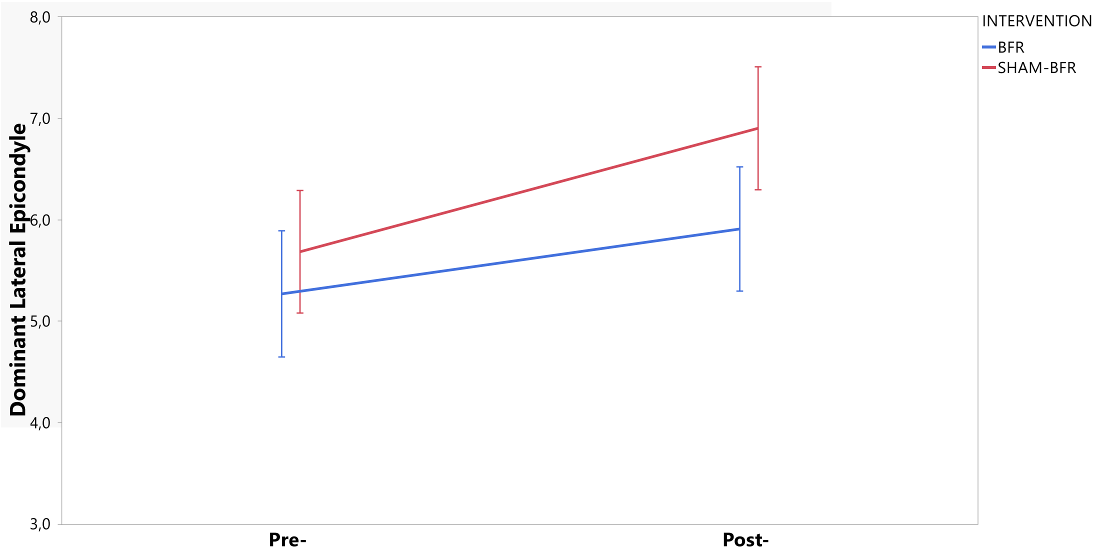

Dominant Lateral Epicondyle
8,0
7,0
6,0
5,0
4,0
3,0
Pre-
Post-
INTERVENTION
BFR
SHAM-BFR

d)

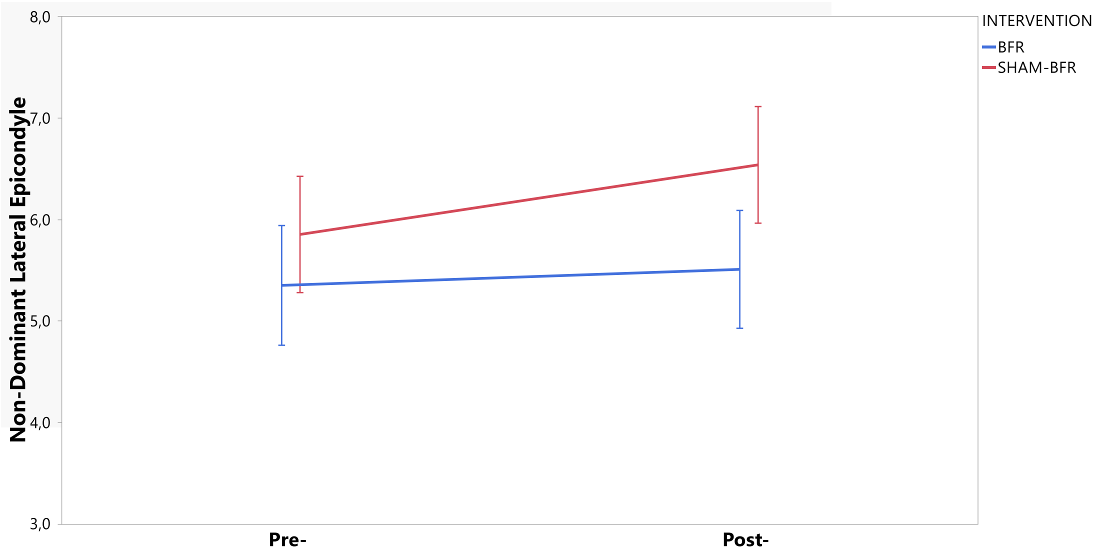

Non-Dominant Lateral Epicondyle
8,0
7,0
6,0
5,0
4,0
3,0
Pre-
Post-
INTERVENTION
BFR
SHAM-BFR

e)

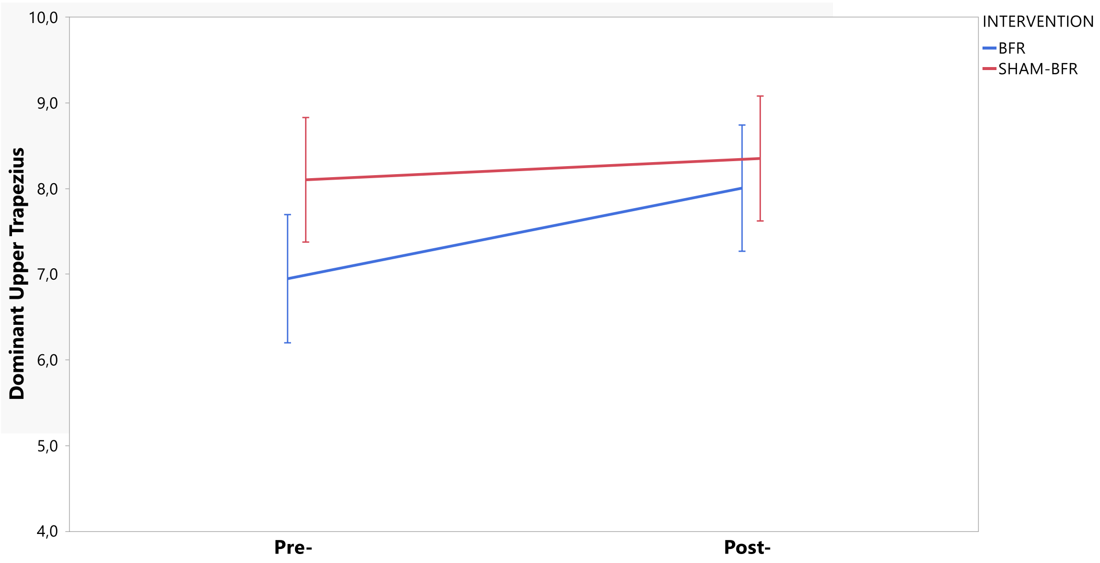
INTERVENTION
BFR
SHAM-BFR
Dominant Upper Trapezius
10,0
9,0
8,0
7,0
6,0
5,0
4,0
Pre-
Post-

f)

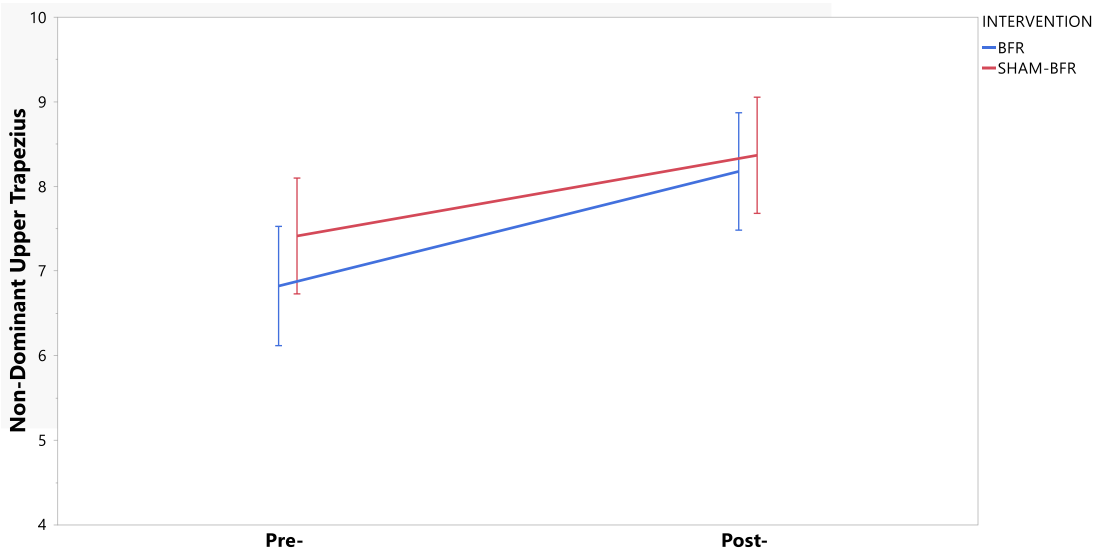
INTERVENTION
BFR
SHAM-BFR
Non-Dominant Upper Trapezius
10
9
8
7
6
5
4
Pre-
Post-

g)

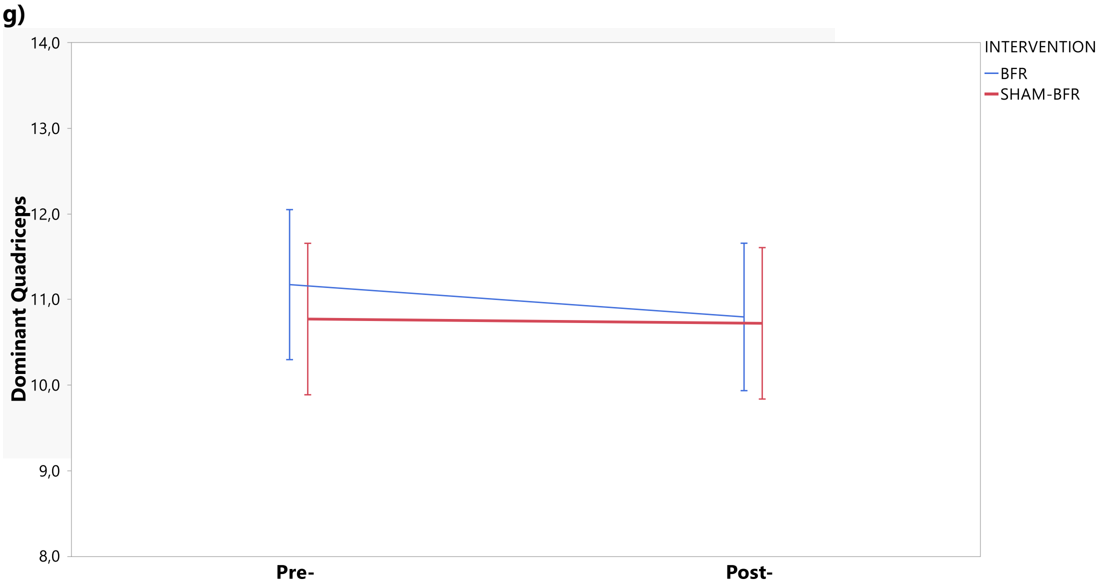

14,0
13,0
12,0
11,0
10,0
9,0
8,0
Dominant Quadriceps
Pre-
Post-
INTERVENTION
BFR
SHAM-BFR

h)

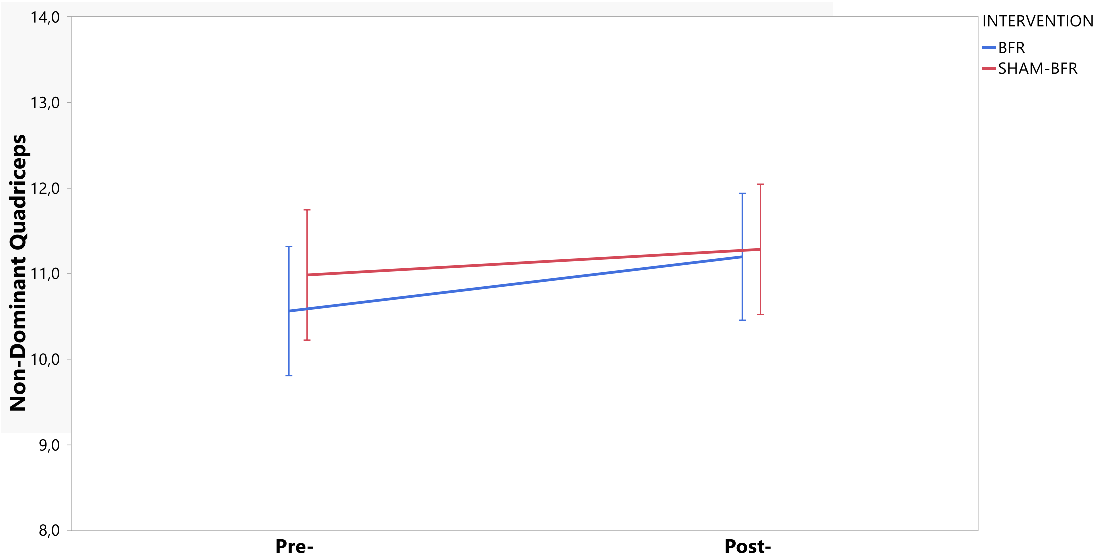

14,0
13,0
12,0
11,0
10,0
9,0
8,0
Non-Dominant Quadriceps
Pre-
Post-
INTERVENTION
BFR
SHAM-BFR
