# Supplementary material for: Exercised-Induced Hypoalgesia following An Elbow Flexion Low-Load Resistance Exercise with Blood Flow Restriction: A Sham-Controlled Randomized Trial in Healthy Adults
Source: Healthcare (Basel). 2022 Dec 16;10(12):2557. doi: 10.3390/healthcare10122557 (PMC9778505; doi:10.3390/healthcare10122557)
Supplement: Supplementary file 1 [file healthcare-10-02557-s001.zip › healthcare-2032975-Supplementary Tables.pdf]

| **Table S1. Baseline pressure pain thresholds values with intra-rater reliability using intraclass correlation coefficients, standard error of measurement and minimum detectable change at each site** | | | | |
|---|---|---|---|---|
| **Assessment site** | **Pressure pain threshold (kg/cm$^2$) ±SD** | **ICC** | **SEM** | **MDC** |
| Dominant Biceps Brachii | 4.98 ±1.8 | 0.95 | 0.4 | 1.11 |
| Non- Dominant Biceps Brachii | 4.9 ±1.7 | 0.93 | 0.44 | 1.24 |
| Dominant Lateral epicondyle | 5.5 ±2.1 | 0.96 | 0.42 | 1.16 |
| Non-Dominant Lateral epicondyle | 5.6 ±2.1 | 0.93 | 0.55 | 1.54 |
| Dominant Upper Trapezius | 7.93 ±3 | 0.99 | 0.3 | 0.83 |
| Non- Dominant Upper Trapezius | 7.3 ±2.3 | 0.98 | 0.32 | 0.9 |
| Dominant Quadriceps | 10.8 ±4 | 0.99 | 0.4 | 1.1 |
| Non-Dominant Quadriceps | 11.5 ±4.1 | 0.93 | 1.08 | 3 |
| **Abbreviations: ICC, intraclass correlation coefficient; SEM, standard error of measurement; MDC, minimal detectable change; kg, kilogram; cm, centimeters; SD, standard deviation** | | | | |

| **Table S2. Coefficients, confidence intervals, and significance statistics for within-participant mediation analyses between exercise intervention ("X"), the change in systolic blood pressure (Mediator 1) and RPE (Mediator 2). The outcome (Y) is the change in pressure pain threshold at local and distal sites of measurement.** | | | |
|---|---|---|---|
| | **Coefficient** | ***p value*** | **95%CI** |
| **Dominant Biceps** | | | |
| Total effect of "*X*" on *Y* | 0.99 | 0.10 | -0.20 to 0.19 |
| Direct effect of "*X*" on *Y* | 1,12 | 0.09 | -,18 to 2,42 |
| Indirect effect (a1*b1, a2*b2)<br>*Systolic Blood Pressure (M1)*<br>*RPE (M2)* | <br>-,0013<br>-,1241 | | <br>-0.30 to 0.19<br>-0.64 to 0.37 |
| **Non- Dominant Biceps** | | | |
| Total effect of "*X*" on *Y* | 0.6095 | 0.34 | -0.68 to 1.9 |
| Direct effect of "*X*" on *Y* | 0.3745 | 0.58 | -1 to 1.75 |
| Indirect effect (a1*b1, a2*b2)<br>*Systolic Blood Pressure (M1)*<br>*RPE (M2)* | <br>0.1345<br>0.1045 | | <br>-0.17 to 0.53<br>-0.29 to 0.68 |
| **Dominant Lateral Epicondyle** | | | |
| Total effect of "*X*" on *Y* | -0.14 | 0.78 | -1.22 to 0.93 |
| Direct effect of "*X*" on *Y* | 0.0409 | 0.84 | -1.1 to 1.18 |
| Indirect effect (a1*b1, a2*b2)<br>*Systolic Blood Pressure (M1)*<br>*RPE (M2)* | <br>0.0629<br>-0.2509 | | <br>-0.1 to 0.43<br>-0.81 to 0.13 |
| **Non-Dominant Lateral Epicondyle** | | | |
| Total effect of "*X*" on *Y* | -0.09 | 0.86 | -1.28 to 1.08 |
| Direct effect of "*X*" on *Y* | -0.2039 | 0.75 | -1.49 to 1.08 |
| Indirect effect (a1*b1, a2*b2)<br>*Systolic Blood Pressure (M1)*<br>*RPE (M2)* | <br>0.2575<br>0.079 | | <br>-0.21 to 0.35<br>-0.41 to 0.61 |
| **Dominant Upper Trapezius** | | | |

| | | | |
|---|---|---|---|
| Total effect of "*X*" on *Y* | 1.3355 | 0.055 | -0.03 to 2.7 |
| Direct effect of "*X*" on *Y* | 0.4760 | 0.42 | -0.73 to 1.68 |
| Indirect effect (a1*b1, a2*b2)<br>*Systolic Blood Pressure (M1)*<br>*RPE (M2)* | <br>0.1313<br>0.7282 | | <br>-0.09 to 0.63<br>-0.008 to 1.61 |
| **Non-Dominant Upper Trapezius** | | | |
| Total effect of "*X*" on *Y* | 1.2653 | 0.054 | -0.03 to 2.56 |
| Direct effect of "*X*" on *Y* | 0.8364 | 0.212 | -0.5 to 2.17 |
| Indirect effect (a1*b1, a2*b2)<br>*Systolic Blood Pressure (M1)*<br>*RPE (M2)* | <br>0.1698<br>0.2590 | | <br>-0.16 to 0.7<br>-0.14 to 0.76 |
| **Dominant Quadriceps** | | | |
| Total effect of "*X*" on *Y* | 1.8421 | 0.026 | 0.23 to 3.45 |
| Direct effect of "*X*" on *Y* | 1.7536 | 0.05 | -0.005 to 3.51 |
| Indirect effect (a1*b1, a2*b2)<br>*Systolic Blood Pressure (M1)*<br>*RPE (M2)* | <br>0.0193<br>0.692 | | <br>-0.56 to 0.46<br>-0.44 to 0.81 |
| **Non-Dominant Quadriceps** | | | |
| Total effect of "*X*" on *Y* | 0.1263 | 0.88 | -1.68 to 1.93 |
| Direct effect of "*X*" on *Y* | 0.1062 | 0.91 | -1.81 to 2.03 |
| Indirect effect (a1*b1, a2*b2)<br>*Systolic Blood Pressure (M1)*<br>*RPE (M2)* | <br>0.2063<br>-0.1863 | | <br>-0.4 to 0.76<br>-1.03 to 0.52 |
| **Abbreviations:** RPE, rating of perceived exertion, CI, confidence interval | | | |

**Table S3. Within-group differences (pre-post) of baseline-adjusted values of pressure pain thresholds and blood pressure.**

| | **LLRE-BFR group<br>Mean (95%CI), Cohen's d<br>p-value** | **HLRE with sham-BFR group<br>Mean (95%CI), Cohen's d<br>p-value** |
|---|---|---|
| **Dominant Biceps** | **-1.61 (-2.37 to -0.85), d=0.88<br>p<0.001** | **0.95 (-1.71 to -0.79), d=0.52<br>p=0.016** |
| **Non-dominant Biceps** | -1.05 (-2.53 to 0.43), d=0.6<br>p=0.25 | -0.57 (-2.05 to 0.91), d=0.33,<br>p=0.74 |
| **Dominant LE** | -0.81 (-2.89 to 1.26), d=0.38<br>p=0.73 | -1 (-3.08 to 1.07), d=0.47<br>p=0.58 |
| **Non-dominant LE** | -0.39 (-2.36 to 1.57), d=0.18<br>p=0.95 | -0.49 (-2.46 to 1.48), d=0.23<br>p=0.98 |
| **Dominant UT** | -1.17 (-3.65 to 1.31), d=0.39<br>p=0.6 | -0.02 (-2.5 to 2.46), d=0.006<br>p=1 |
| **Non-dominant UT** | -1.47 (-3.8 to 0.86), d=0.63<br>p=0.35 | -0.75 (-3.08 to 1.57), d=0.32<br>p=0.82 |
| **Dominant Quadriceps** | 0.005 (-2.94 to 2.95), d=0.0001<br>p=1 | 0.38 (-2.64 to 3.41), 0.09<br>p=0.98 |
| **Non-dominant Quadriceps** | -0.99 (-3.65 to 1.66), d=0.24<br>p=0.75 | 0.07 (-2.65 to 2.8), d=0.01<br>p=0.99 |
| **Systolic Blood Pressure** | 4.8 (-9.18 to 18.78), d=0.25<br>p=0.8 | -4.8 (-19 to 9.28), d=0.25<br>p=0.51 |

| | | |
|---|---|---|
| **Diastolic Blood Pressure** | 0.1 (-6.3 to 6.5), d=0.01<br>p=1 | -0.38 (-6.8 to 6.1), d=0.04<br>p=0.92 |

Note: Adjustments were performed for post-hoc multiple comparisons (Bonferroni)

**Abbreviations:** BFR, blood flow restriction; LLRE-BFR; low load resistance exercise with blood flow restriction; LE, lateral epicondyle; UT, upper trapezius; SD, standard deviation; CI, confidence interval
